# Mechanisms of Immunomodulation and Cytoprotection Conferred to Pancreatic Islet by Human Amniotic Epithelial Cells

**DOI:** 10.1007/s12015-021-10269-w

**Published:** 2021-10-06

**Authors:** Fanny Lebreton, Reine Hanna, Charles H. Wassmer, Kevin Bellofatto, Lisa Perez, Véronique Othenin-Girard, Begoña Martinez de Tejada, Marie Cohen, Ekaterine Berishvili

**Affiliations:** 1 Laboratory of Tissue Engineering and Organ Regeneration, Department of Surgery, University of Geneva, Geneva, Switzerland; 2 Cell Isolation and Transplantation Center, Department of Surgery, Geneva University Hospitals and University of Geneva, Geneva, Switzerland; 3 Diabetes Center of the Faculty of Medicine, University of Geneva, Geneva, Switzerland; 4 Department of Pediatrics, Gynecology and Obstetrics, Geneva University Hospitals, Geneva, Switzerland; 5 School of Natural Sciences and Medicine, Ilia State University, Tbilisi, Georgia; 6 Faculty of Medicine, University of Geneva, 1211 Geneva, Switzerland

**Keywords:** Human amniotic epithelial cells, Immonomodulation, Cytoprotection, Pancreatic islets, Pro-inflammatory cytokines

## Abstract

Inhibiting pro-inflammatory cytokine activity can reverse inflammation mediated dysfunction of islet grafts. Human amniotic epithelial cells (hAECs) possess regenerative, immunomodulatory and anti-inflammatory properties. We hypothesized that hAECs could protect islets from cellular damage induced by pro-inflammatory cytokines. To verify our hypothesis, hAEC monocultures, rat islets (RI), or RI-hAEC co-cultures where exposed to a pro-inflammatory cytokine cocktail (Interferon γ: IFN-γ, Tumor necrosis factor α: TNF-α and Interleukin-1β: IL-1β). The secretion of anti-inflammatory cytokines and gene expression changes in hAECs and viability and function of RI were evaluated. The expression of non-classical Major Histocompatibility Complex (MHC) class I molecules by hAECs cultured with various IFN-γ concentrations were assessed. Exposure to the pro-inflammatory cocktail significantly increased the secretion of the anti-inflammatory cytokines IL6, IL10 and G-CSF by hAECs, which was confirmed by upregulation of IL6, and IL10 gene expression. HLA-G, HLA-E and PDL-1 gene expression was also increased. This correlated with an upregulation of STAT1, STAT3 and NF-κB1gene expression levels. RI co-cultured with hAECs maintained normal function after cytokine exposure compared to RI cultured alone, and showed significantly lower apoptosis rate. Our results show that exposure to pro-inflammatory cytokines stimulates secretion of anti-inflammatory and immunomodulatory factors by hAECs through the JAK1/2 – STAT1/3 and the NF-κB1 pathways, which in turn protects islets against inflammation-induced damages. Integrating hAECs in islet transplants appears as a valuable strategy to achieve to inhibit inflammation mediated islet damage, prolong islet survival, improve their engraftment and achieve local immune protection allowing reducing systemic immunosuppressive regimens.

**Graphical Abstract:**

This study focuses on the cytoprotective effect of isolated hAECs on islets exposed to pro-inflammatory cytokines in vitro. Exposure to pro-inflammatory cytokines stimulated secretion of anti-inflammatory and immunomodulatory factors by hAECs putatively through the JAK1/2 – STAT1/3 and the NF-κB1 pathways. This had protective effect on islets against inflammation-induced damages. Taken together our results indicate that incorporating hAECs in islet transplants could be a valuable strategy to inhibit inflammation mediated islet damage, prolong islet survival, improve their engraftment and achieve local immune protection allowing to reduce systemic immunosuppressive regimens.

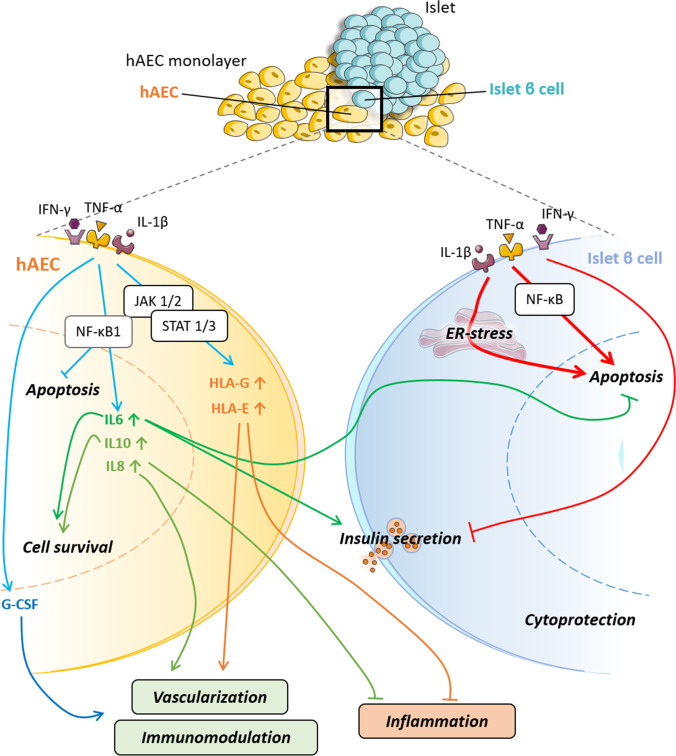

## Introduction

Islet transplantation is a minimally invasive approach allowing restoring glycemic control in diabetic patients [56]. However, long-term function of the graft and steady metabolic control remain a challenge due to ischemic, inflammatory, allogenic and autoimmune attacks causing damages to the transplanted islets [13]. Significant number of islets are destroyed immediately after transplantation, due to the onset of inflammatory reaction [24]. Inflammatory islet damage is mediated at least partially by pro‐inflammatory cytokines, such as interferon-γ (IFN-γ), tumor necrosis factor-α, (TNF-α) and interleukin 1β (IL-1β) [3]. When exposed to these pro-inflammatory cytokines, both rodent and human islet cells lose ability to respond to glucose stimulation and undergo apoptosis [9]. Moreover, these cytokines recruit and activate macrophages, thus aggravating the inflammatory response [24]. It has been shown that decreasing cytokine expression, and inhibiting cytokine and macrophage activity significantly improves the function of transplanted islets [26]. Therefore, minimizing inflammation at the transplant site has been considered a major strategy to prolong islet graft function and maintain long-term insulin independence [10].

Among several approaches proposed to improve islet transplantation outcomes, islet co-transplantation with accessory non islet-derived cells showed promising experimental results [58]. Mesenchymal stem cells (MSCs) have been the main cell types used to protect islets from inflammation injury due to their anti-inflammatory and/or immunomodulatory properties. Several studies have shown that co-culturing islets with MSCs derived from different sources (adipose tissue or bone marrow) protected islets from inflammation and enhanced their revascularization, function and engraftment [1]. Over the last decades, MSCs have been intensively used in regenerative medicine, especially for the treatment of inflammatory and degenerative disorders [52]. However, harvesting MSCs is an invasive procedure and obtaining sufficient number of cells is not always possible, as cell numbers and properties decline with donor age. Tumorigenicity of MSCs is also a concern [19]. Among the several cell sources available for tissue regeneration, human amniotic epithelial cells (hAECs) have been recognized as valid candidates [38, 53]. They reside on the amniotic membrane and together with other placental cells are believed to participate to the development of materno-fetal tolerance during pregnancy [57]. hAECs express nuclear markers of pluripotency and surface markers of embryonic stem cells (ESC) such as SSEA-4, OCT-4 and SOX-2 [44]. When grown in specialized media, hAECs differentiate toward all three germ layers [21]. Furthermore, hAECs exhibit anti-inflammatory and immunomodulatory properties, are easily accessible, inexpensive and do not bear the risk of tumorigenicity [57]. Several in vitro and in vivo studies have demonstrated pleiotropic immune regulatory activities of hAECs, mediated by complex mechanisms that inhibit the function of different cell subpopulations of innate and adaptive immunity [38]. hAECs express low levels of major histocompatibility complex (MHC) class I surface antigens, while MHC class II antigens [2], or the costimulatory molecules CD80, CD86, CD40, and CD154 are not expressed, even in the presence of IFN-γ [2, 27]. hAECs inhibit local activation/migration of neutrophils and macrophages and suppress the activation and cytotoxic action of T-cells in both mixed lymphocyte reaction and mitogen-induced proliferation assays [2]. Expression and secretion of several mediators of localized immune suppression including TGFβ, HLA-G, IL-6, IL-10 and Fas-L have been identified in isolated hAECs [20, 34]. The anti-inflammatory, immunomodulatory, and cytoprotective effects of hAECs have been validated in vivo on different preclinical models, including, liver fibrosis [33, 39, 40], lung fibrosis [4–6, 54], wound healing [61], and inflammatory bowel disease [47]. Application of hAECs was associated with the reduction of neutrophil, macrophage, monocyte and T-cell infiltration. Inflammation-related cytokines were also reduced [38]. Furthermore, it has been shown that in vitro exposure of hAECs to IFN-γ significantly increases the expression of immunomodulatory molecule, such as HLA-G and Programmed Death Ligand 1 (PD-L1) [29, 30].

Our group has studied the effect of hAECs on islet cells. We have recently shown that either combining hAECs with dissociated islet cells in insulin‐secreting organoids or shielding of whole islets with hAECs protects islet cells against hypoxia‐induced damage in vitro and improves β cell engraftment and vascularization after transplantation in diabetic mice [31, 32]. These characteristics indicate that hAECs may be capable of creating a microenvironment conducive to sustained islet graft survival.

In this study, we have examined the cytoprotective effect of isolated hAECs on islets exposed to pro-inflammatory cytokines and established some of the underlying mechanisms.

## Materials and Methods

### Human Amniotic Epithelial Cells (hAECs) Isolation and Characterization

Experiments using human tissues were covered by protocols approved by the state of Geneva Ethical Committee (Commission Cantonale d’Ethique de la Recherche – CCER). Amniotic membranes were harvested from term healthy placentas of women undergoing elective cesarean section at the Geneva University Hospitals. Informed written consent was obtained from each donor prior to tissue collection.

hAECs were isolated as previously described [31]. Briefly, the amniotic membrane was mechanically peeled from the underlying chorion, washed in Hanks' Balanced Salt Solution (HBSS, ThermoFisher Scientific, Reinach, Switzerland), cut into small pieces and trypsinised (0.25% Trypsin/EDTA, ThermoFisher Scientific) to release cells. Dispersed hAECs were collected by centrifugation, seeded at a density 2 × 10^5^ cells/cm^2^ and cultured in hAEC culture medium consisting in DMEM/F-12 medium (ThermoFisher Scientific,) supplemented 2 mmol/l L-Glutamin, 100 U/ml Penicillin and 0.1 mg/ml Streptomycin (1% (v/v) of a L-Glutamin-Penicillin–Streptomycin stock solution from Sigma-Aldrich), 1 mmol/l sodium pyruvate (Sigma-Aldrich), 1% (v/v) MEM NEAA 100X (ThermoFisher Scientific), 0.1% fungin (InvivoGen, San Diego, CA), 10% fetal bovine serum (FBS; Merk Millipore, Zug, Switzerland), 0.05 mmol/l 2-mercaptoethanol (ThermoFisher Scientific), 10 ng/ml human recombinant epidermal growth factor (EGF, Sigma-Aldrich).

**Fig. 1:**
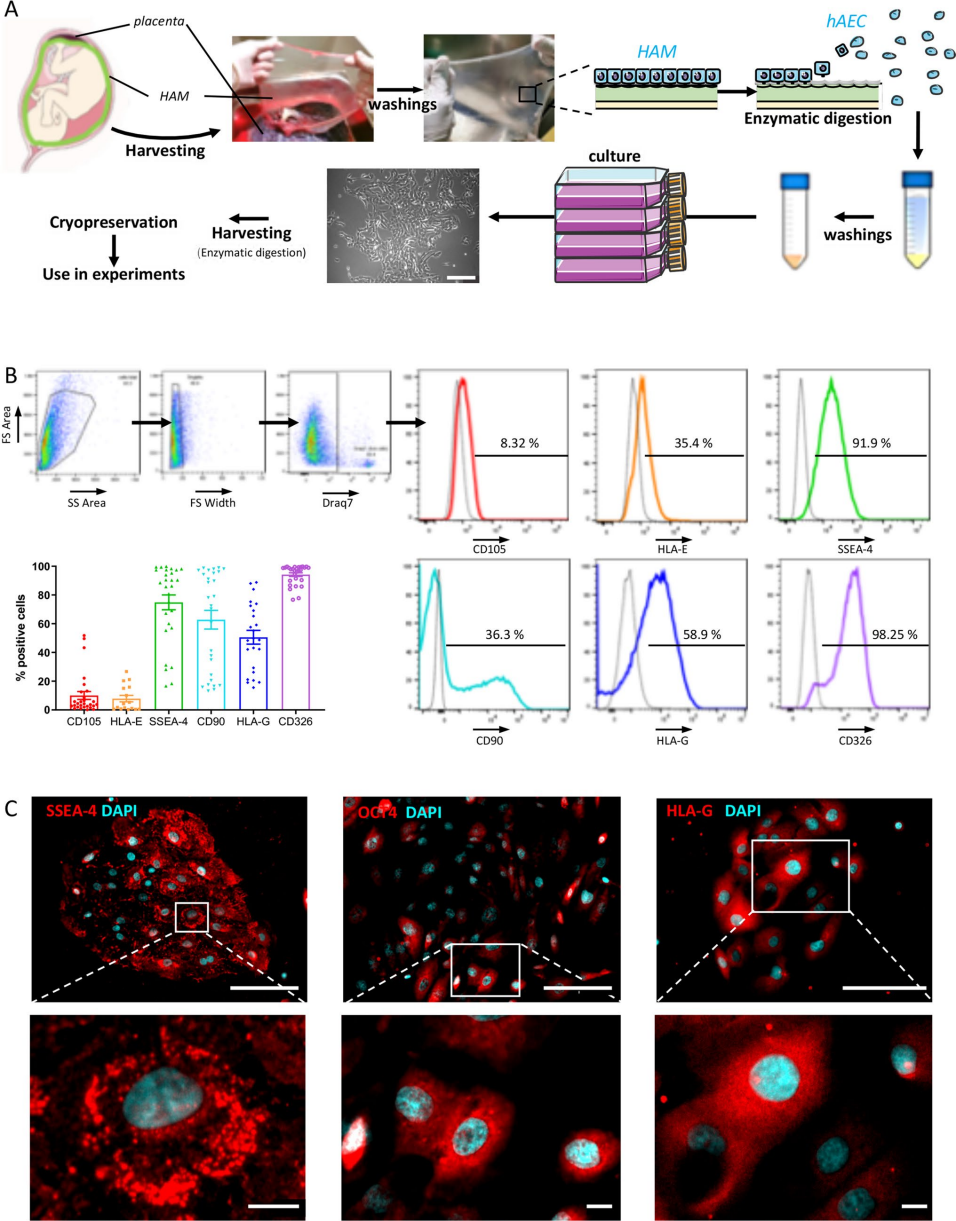
Isolation and characterization of human amniotic epithelial cells (hAECs). **A** Schematic representation of hAEC isolation protocol. Human amniotic membrane is harvested from term placenta, washed and hAECs are detached from the membrane using trypsin–EDTA. The resulting cells are then washed and cultured for 5–7 days. HAECs are then removed from the culture flasks at 80% confluence by mild trypsinization, and cryopreserved for later use. **B** hAECs were characterized by flow cytometry. Upper left panel shows the gating strategy used to obtain the quantification of positive populations for mesenchymal markers (CD105, CD90), pluripotency marker (SSEA-4), epithelial marker (CD326) and immunomodulatory markers (HLA-E and HLA-G). Representative examples are shown in the right panel, and quantification from 28 distinct hAEC batches (i.e. isolated from 28 placentae) is shown in the bottom left panel. **C** Immunofluorescent images of hAECs stained for the pluripotency markers SSEA-4 and Oct-4 and the immunomodulatory marker HLA-G (upper panels, scale bars = 100 um) and corresponding magnifications (lower panels, scale bars = 10 um)

Confluent cells were trypsinised (0.05% Trypsin/EDTA, ThermoFisher Scientific) and characterized by flow cytometry (FC; Fig. 1A). Cells were washed in FC buffer (PBS-0.1% BSA supplemented with 0.01% sodium azide) and incubated for 30 min at 4 °C with the following antibodies: FITC-conjugated anti-human CD105 (clone 266), BV421-conjugated anti-human CD326 (clone EBA-1), PerCP-Cy5.5 conjugated anti-SSEA4 (clone MC813-70) (1:50 dilution; all from BD Biosciences, Allschwil, Switzerland), PE-Cy7 conjugated anti-human CD90 (clone 5E10) (1:100 dilution, BD Biosciences), PE-conjugated anti-human HLA-E (clone 3D12) and APC-conjugated anti-human HLA-G (clone 87G) (1:20 dilution, Biolegend, London, UK) antibodies. Controls were stained with isotype-matched irrelevant antibody to evaluate non-specific binding to target cells. Cells were analyzed on a Gallios cytometer (Beckman Coulter, Indianapolis, Indiana, US) using Kaluza Analysis software from Beckman Coulter (Version 1.5.20365.16139). The percentage of positive cells for the different markers was assessed on a gate set on hAECs by using forward- and side-scatter analysis during the acquisition of data, followed by doublets and Draq7-positive (dead) cells exclusion. Representative histograms were plotted using FlowJo software (version 10.6.1, BD Biosciences). hAECs cultured on collagen-coated coverslips were fixed in 4% paraformaldehyde (PFA), rinsed and permeabilized. For histological characterization, after two washes, unspecific binding sites were blocked and samples were stained with the following primary antibodies: monoclonal anti-SSEA-4 (1:75 dilution, clone MC813, Abcam, Cambridge, UK), polyclonal anti-Oct4 (1:200 dilution, Abcam, Cambridge, UK) and monoclonal anti-human HLA-G (1:50 dilution, clone 4H84, BD Biosciences). The secondary antibodies were Alexa 555 anti-mouse or anti-rabbit antibodies (1:300 dilution, ThermoFisher Scientific). Stained cells were mounted with aqueous mounting medium containing DAPI (Fluoroshield Mounting Medium with DAPI, Abcam). Images were captured using a Zeiss Axioscan.Z1 slide scanner (Zeiss, Feldbach, Germany).

### Rat Pancreatic Islets Isolation

Animal experiments were performed under protocols reviewed and approved by the Geneva Veterinary authorities and the University of Geneva Institutional Animal Care and Use Committee.

Ten-week old male Lewis rats (LEW/OrlRj; Janvier Labs, Le Genest St-Isle, France) were used for pancreatic islet isolation. Rat pancreatic islets were isolated as previously described [22, 23], purified by Ficoll density centrifugation and cultured in islet culture medium consisting of DMEM (ThermoFisher Scientific) supplemented with 10% (v/v) FBS (ThermoFisher Scientific), 1 mmol/l sodium pyruvate, 11 mmol/l glucose (Bichsel, Interlaken, Switzerland), 0.05 mmol/l 2-mercaptoethanol, 2 mmol/l L-Glutamine, 100 U/ml Penicillin and 0.1 mg/ml Streptomycin for one day prior to be used in co-culture experiments.

### Exposure of hAECs to IFN-γ

hAECs from 4 distinct placentas were used in this experiment. hAECs were seeded onto 25cm^2^ tissue culture-treated flasks at a density of 17′500 cells/cm^2^. Culture media were replaced the next day and cells were exposed to various concentrations of recombinant murine Interferon-gamma (IFN-γ, Peprotech, London, UK, reference 315–05) for 48 h. Untreated hAECs served as controls. Supernatants from treated and untreated hAECs were analyzed to detect secreted anti- and pro-inflammatory factors. hAEC phenotype were assessed by FC analysis.

### Exposure of hAECs: Islet Co-culture to Pro-inflammatory Cytokines

hAECs were thawed and added (1.0 × 10^6^ cells) to a 100 mm tissue culture-treated dish with 500 islet equivalents (IEQ) in a total volume of 10 mL hAEC culture medium. Rat islet (RI) and hAEC monocultures were used as controls. After 24 h incubation, a pro-inflammatory cytokine cocktail consisting of 100 U/mL recombinant murine IFN-γ (reference 315–05), 800 U/mL recombinant human Tumor Necrosis Factor-alpha (TNF-α, reference 300-01A) and 50 u/mL recombinant human Interleukine-1 beta (IL-1β, reference 200-01B) were added to 10 ml of culture medium for an additional 48 h (Fig. 3A).

All cytokines were obtained from Peprotech (London, UK). 6 different hAEC preparations were used in this experiment.

### Analysis of Culture Supernatant for Soluble Cytoprotective Factors

Culture supernatants from hAEC monocultures were sampled before and after cytokine exposure and stored at -20 °C until further assessment. Qualitative detection of secreted anti- and pro-inflammatory factors (IL6, IL10, TNF-α, G-CSF and TGF-β1) in the culture media was performed using the commercial Multi-Analyte ELISArray assay MEH003A from Qiagen (Courtaboeuf, France). Released IL6, IL10 and G-CSF were quantified using Quantikine ELISA for human IL-6, IL-10 and G-CSF (R&D Systems, Abingdon, UK), according to manufacturer instructions.

### Cell Apoptosis Assay

RI (100 IEQ), hAECs (2 × 10^5^ cells) and RI + hAECs (2 × 10^5^ hAECs + 100 IEQ) cultured in 35 mm^2^ petri dishes with or without cytokines were washed with PBS and cytoplasmic histone-associated DNA fragments were extracted and quantified using the Cell Death Detection ELISA kit from Roche (Sigma-Aldrich) according to manufacturer instructions.

### Islet Functional Assay

RI and RI + hAECs cultured with or without cytokines were assessed in duplicates for glucose stimulated insulin secretion. After a 1 h pre-incubation in Krebs–Ringer buffered HEPES (pH 7.4) with 0.1% (w/v) BSA (KRB) at 37 °C, islets and cells were successively incubated for 1 h at 37 °C in KRB solutions containing glucose at low (2.8 mmol/l) or high (16.7 mmol/l) concentration. Total insulin content was extracted using acid–ethanol solution. Supernatants were collected after each incubation time, insulin concentration was determined by ELISA (Mercodia, Uppsala, Sweden) and normalized to the total insulin content of the corresponding lysates. Islet responsiveness to glucose was defined as the ratio of insulin secretion in high glucose to insulin secretion in low glucose solution, hereafter referred to as the stimulation index (SI).

### Real-Time Quantitative Polymerase Chain Reaction

RNA was extracted from hAEC monocultures (10^6^ cells in 10 cm^2^ petri dishes) or RI + hAEC co-cultures using the RNeasy minikit (Qiagen, Courtaboeuf, France) after 48 h of inflammatory cytokine cocktail exposure. cDNAs were synthesized by reverse-transcription using the GoScriptTM Reverse Transcription Kit (Promega, Dübendorf, Switzerland). RT-qPCR was performed using the Takyon No-Rox SYBR Core Kit blue dTTP (Eurogentec, Liège, Belgium), or the Taqman Fast Advance Master Mix (Thermofisher Scientific). Primers used for amplification were purchased from Microsynth (Balgach, Switzerland) and Thermofisher Scientific and targeted the following genes: human *IL4, IL6, IL8, IL10, HLA-G, HLA-E, STAT1, STAT3, JAK1, JAK2 NFKB1*, and rat *Bcl2* and *Nfkb1.* Gene expression values were normalized to the housekeeping genes human *RPLP0*, human *EIF2B1*, rat *Rplp1* and rat *Actb,* and calculated with the comparative cycle threshold Ct method (2-ΔCt method). All primer forward and reverse sequences are detailed in Table 1.

**Table 1:** List of the genes evaluated for expression changes by RT-qPCR and associated primer forward and reverse sequences

Species	Gene	Forward sequence	Reverse sequence	Method
Human	*IL4*	ACT GCA CAG CAG TTC CAC AG	CTC TGG TTG GCT TCC TTC AC	SYBR
Human	*IL6*	CAC ACA GAC AGC CAC TCA CC	TTT TCT GCC AGT GCC TCT TT	SYBR
Human	*IL8*	GAT CCA CAA GCC TTG TTC	CGT AAT TCA ACA CAG CAC TAC	SYBR
Human	*IL10*	AAG CCT GAC CAC GCT TTC TA	GCT CCC TGG TTT CTC TTC CT	SYBR
Human	*HLA-G*	TTG CTG GCC TGG TTG TCC TT	TTG CCA CTC AGT CCC ACA CAG	SYBR
Human	*HLA-E*	CCT ACG ACG GCA AGG A	CCC TTC TCC AGG TAT TTG TG	SYBR
Human	*STAT1*	TTC AGG AAG ACC CAA TCC AG	TGA ATA TTC CCC GAC TGA GC	SYBR
Human	*STAT3*	AGT GAG TAA GGC TGG GCA GA	AAG GCA CCC ACA GAA ACA AC	SYBR
Human	*JAK1*	CGC TCT GGG AAA TCT GCT AC	AGG TCA GCC AGC TCC TTA CA	SYBR
Human	*JAK2*	GAG CCT ATC GGC ATG GAA TA	TTA TCC ATC CGT GCA CAA AA	SYBR
Human	*NFKB1*	AAC AGA GAG GAT TTC GTT TCC G	TTT GAC CTG AGG GTA AGA CTT CT	SYBR
Human	*RPLP0*	AAG GTG TAA TCC GTC TCC ACA GA	TGC ATC AGT ACC CCA TTC TAT CAT	SYBR
Human	*EIF2B1*	CGG ACG TTG CTG GAG TTC TT	CCA CAC CAC ACA GGG TTT CT	SYBR
Rat	*Nfkb1*	GGG CTG ACC TGA GTC TTC TG	GAT AAG GAG TGC TGC CTT GC	SYBR
Rat	*Rplp1*	TCT CTG AGC TTG CCT GCA TCT ACT	CCT ACA TTG CAG ATG AGG CTT CCA	SYBR
Rat	*Bcl2*	Rn99999125-m1		Taqman
Rat	*Actb*	Rn00667869-m1		Taqman

### Statistical Analysis

Continuous and categorical variables are presented as mean ± SEM. Comparisons between groups were performed with unpaired two-tailed Student’s *t-*test or one-way / two-way ANOVA with Tukey’s or Sidak’s post-hoc test wherever appropriate. All statistical analyses were performed with Prism software 7.02 (GraphPad, La Jolla, CA, USA), and p < 0.05 was considered statistically significant.

## Results

### Characterization of hAECs

hAECs isolated following a standardized protocol (Fig. 1A) were characterized using FC (Fig. 1B). hAECs were positive for CD326, CD90, CD105, SSEA-4, HLA-G and HLA-E. Expression of the pluripotency and immunomodulatory markers was confirmed by immunohistological staining (Fig. 1C), showing the cytosolic localization of SSEA-4 and HLA-G, while OCT4 was found in both the cytoplasm and the nucleus. These findings are in line with our and other’s previous findings [31, 32, 57].

### IFN-γ Augments Immunomodulatory Potential of hAECs

**Fig. 2:**
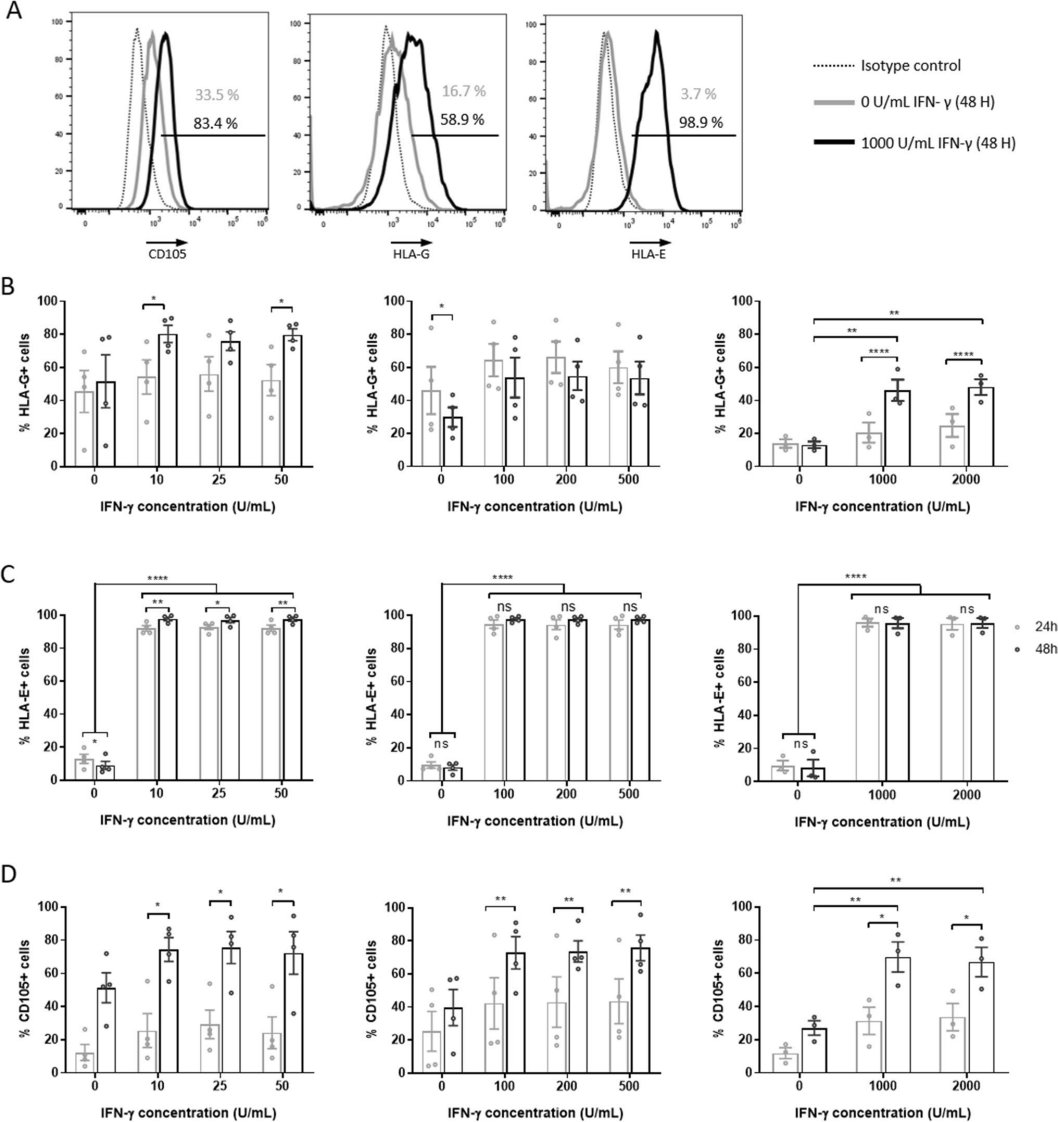
IFN-γ increases the expression of HLA-E and HLA-G in hAECs. hAEC monocultures were exposed to various concentration of IFN-γ for 24 and 48 h and were characterized by flow cytometry. **A** Representative flow cytometry histograms for CD105 (left panel), HLA-G (central panel) and HLA-E (right panel) positive populations in hAEC cultures exposed for 48 h to 1000 U/mL IFN-γ (dark lines) compared to untreated hAECs (grey lines). Isotype control is shown as dotted lines. Positive population for HLA-G (**B**), HLA-E (**C**) and CD105 (**D**), were quantified after exposition to low (left panels: 10, 25 and 50 U/mL, n = 4), medium (central panels: 100, 200 and 500 U/mL, n = 4) and high (right panels: 1000 and 2000 U/mL, n = 3) concentrations of IFN-γ. Grey bars: 24 h exposure, black bars: 48 h exposure. * p < 0.05, ** p < 0.01, **** p < 0.0001

In the initial experiment, we have assessed whether exposure to IFN-γ at various concentrations had an impact on immunomodulatory and other markers expressed by hAECs (Fig. 2).

FC analysis demonstrated that HLA-G expression was not affected by low concentrations of IFN-γ, but was significantly increased by both 1000 U/mL and 2000 U/mL (46.1 ± 6.5% and 48.1 ± 4.8% respectively) IFN-γ treatments compared to untreated cells (13.1 ± 1.9%) (p = 0.0048 and p = 0.0011 respectively). Interestingly, the increase in HLA-G expression was correlated with time in culture (Fig. 2B). Expression of HLA-E was significantly increased in primed cells. More than 90% of treated cells were expressing HLA-E on their surface and the lowest concentration of the cytokine was sufficient to affect this marker (p < 0.0001) (Fig. 2C).

Treated cells expressed CD90, CD326 and SSEA-4 without alterations. In contrast, IFN-γ had an impact on the expression of CD105 (Fig. 2D) in a dose-dependent manner. Although expression of CD105 remained unchanged at low IFN-γ concentrations, exposure of cells to higher concentrations (1000 U/ml and 2000 U/mL) significantly increased CD105 expression compared to untreated controls (Fig. 2D).

### In Vitro Exposure to Pro-inflammatory Cytokines Enhances Anti-inflammatory and Immunomodulatory Properties of hAECs

**Fig. 3:**
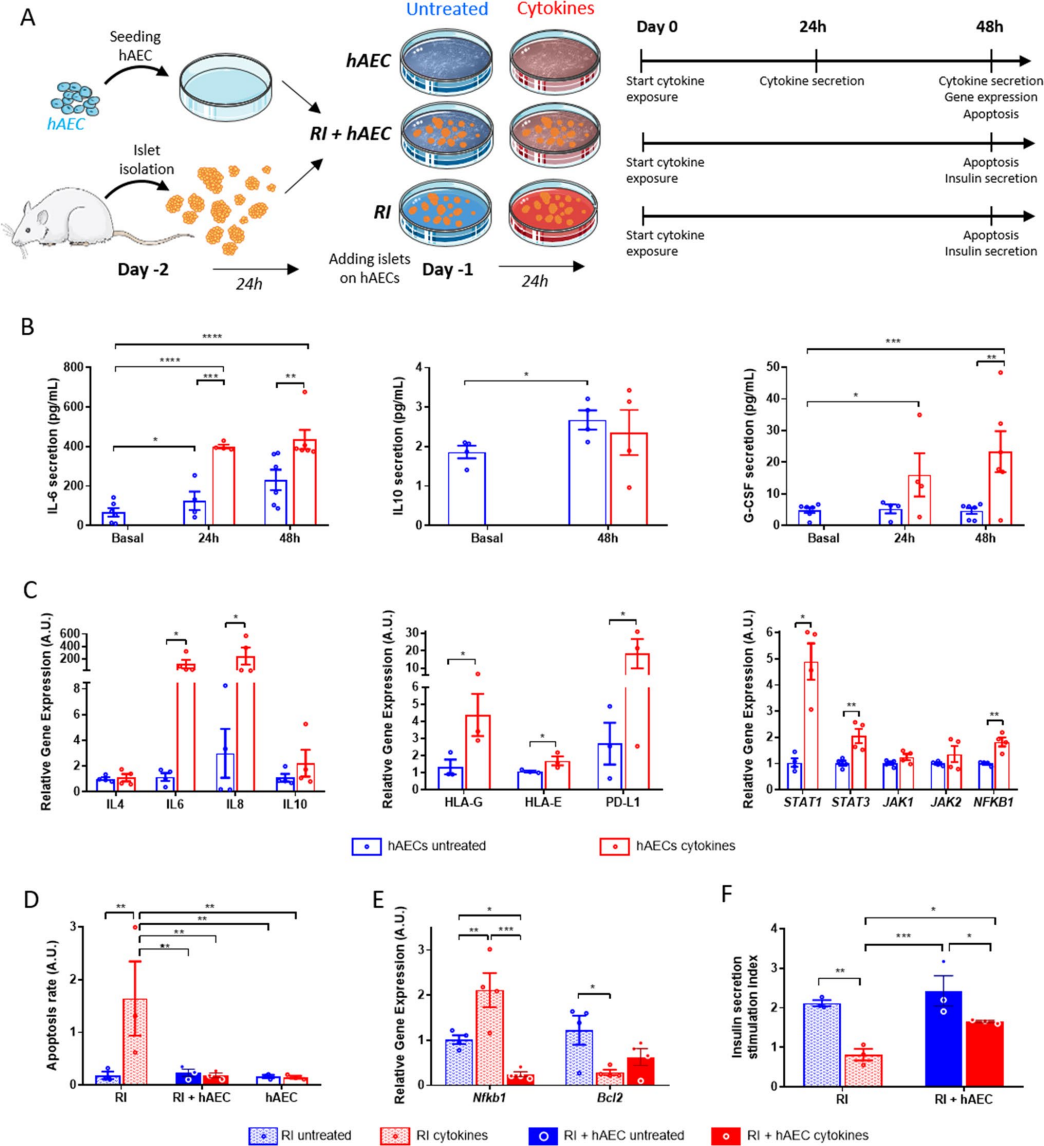
hAECs secrete anti-inflammatory cytokines under pro-inflammatory conditions and protect rat islets against proinflammatory cytokine-induced damages in vitro. **A** Schematic representation of the experimental protocol. hAECs were cultured for 48 h before being exposed to a pro-inflammatory cytokines cocktail (100 U/mL IFN-γ, 800 U/mL TNF-α,50 u/mL IL-1β) for 48 h. Rat islets (RI) were added 24 h after hAEC seeding and were cultured on the hAEC monolayers for 24 h before cytokines exposure. Controls were untreated and cytokines-exposed RI monocultures. **B** IL6 (left, n = 6), IL10 (central, n = 4) and G-CSF (right, n = 6) secretion quantifications in the hAEC culture supernatants before, after 24 h and 48 h of cytokines cocktail exposure. **C** Expression changes in hAEC monocultures after cytokines cocktail exposure for anti-inflammatory cytokine genes (left), immunomodulatory genes (central) and IFN-γ signaling-related genes (right) (n = 4). **D** Apoptosis rate (*i.e.* relative quantification of cytoplasmic histone-associated DNA fragments) in RI and hAEC monocultures and in RI + hAEC co-cultures untreated or exposed to the cytokines cocktail for 48 h (n = 3). **E** Expression changes in rat islets cultured alone or with hAECs after cytokines cocktail exposure for the pro-apoptotic *Nfkb1* and the anti-apoptotic *Bcl2* genes (n = 4). **F** Islet function given as the secretion index during a glucose stimulated insulin secretion test performed on RI cultured alone or with hAECs in the presence or absence of pro-inflammatory cytokines (n = 3). Blue bars: Untreated cultures, red bars: cultures exposed to the cytokine cocktail. Empty bars: hAEC monocultures, patterned bars: RI monocultures, filled bars: RI + hAEC cocultures. * p < 0.05, ** p < 0.01, *** p < 0.001, **** p < 0.0001

β cell death associated with nonspecific inflammation is mainly mediated by production of pro‐inflammatory cytokines such as IFN-γ, TNF-α and IL-1β [3, 43]. In order to examine whether these inflammatory cytokines would have an impact on anti-inflammatory and immunomodulatory potential of hAECs, cells were treated with the three-cytokine cocktail. Detailed experimental design is shown in Fig. 3A.

Basal levels of cytokines secreted by hAECs (control) were measured in the supernatants after 24 h of culture. Supernatants from hAECs exposed to cytokines were analyzed after 24 h and 48 h of incubation. Although baseline levels of IL6, TNFα and TGF-β1 were quite low in control samples, a significant increase in IL6 concentration was detected in supernatants from primed cells both at 24 h and 48 h after the cytokines treatment (p < 0.0001 vs control at both time points). TNFα and G-CSF concentration were also elevated (data not shown).

IL6 and G-CSF concentrations were also measured by ultra-sensitive ELISA assay (Fig. 3B). Concentrations of IL6 in the culture medium of treated cells were significantly higher compared to untreated controls both at 24 h (400.9 ± 10.1 pg/mL, vs 125.1 ± 47.1 pg/mL, p = 0.0005) and 48 h (435.8 ± 48.5 pg/mL, vs 231.4 ± 51.9 pg/mL, p = 0.0015). Although IL10 was detectable in the control samples and its concentration increased between 24 and 48 h of culture, no significant effect of cytokines exposure was observed. In contrast, G-CSF secretion was significantly elevated both after 24 h (16.0 ± 6.8 pg/mL vs 4.8 ± 0.9 pg/mL, p = 0.0196) and 48 h (23.4 ± 6.4 pg/mL vs 4.8 ± 0.9 pg/mL, p = 0.0003) compared to basal level, as well as in comparison with untreated culture at 48 h (23.4 ± 6.4 pg/mL and 4.7 ± 0.97 pg/mL respectively, p = 0.0024).

To reveal the mechanisms behind the anti-inflammatory action of hAECs, gene expression changes were evaluated (Fig. 3C). Pro-inflammatory cytokines did not evidently affect IL4 gene expression. In contrast, exposure of cells to pro-inflammatory conditions significantly upregulated IL6 and IL8 genes (113.9 ± 69.7 and 81.6 ± 73.2 fold, respectively). Moreover, two-fold upregulation of IL10 was detected compared to control. Similarly, mRNA levels of HLA-G, HLA-E and PD-L1 were significantly upregulated.

Changes in expression of genes involved in intracellular signaling cascade were also studied. STAT1, STAT3 and NF-κB1 were significantly overexpressed in treated hAECs compared to controls, with a 4.9 ± 0.69, 2.06 ± 0.3 and 1.83 ± 0.2 fold increase respectively. JAK1 and JAK2 exhibited a trend to slightly increase.

These data indicate that exposure of primary hAECs to inflammatory cytokines, IFN- γ, TNF-α and IL-1β promotes their anti-inflammatory properties and increases expression of immunomodulatory molecules.

### hAECs Co-cultured with Islets Prevent Cytokine Induced Islet Cell Apoptosis and Preserve Islet Function Under Exposure to Cytokines

To investigate whether anti-inflammatory factors secreted by hAECs in response to pro‐inflammatory cytokines would be able to protect islets against inflammation-induced damage, RI + hAEC co-cultures were exposed to the three-cytokine cocktail (Fig. 3A). The islet cell damage was assessed by quantification of cytoplasmic histone-associated DNA fragments in culture lysates. RI exposed to cytokines showed significant increase in the apoptotic rate compared to untreated controls (1.64 ± 0.7 A.U. and 0.18 ± 0.07 A.U. respectively, p = 0.0041). In contrast, islets co-cultured with hAECs had an apoptosis rate similar to intact controls (0.19 ± 0.05 A.U.) (Fig. 3D). These results correlated with a significant upregulation of the pro-apoptotic gene NF-κB1 (2.11 ± 0.39 fold, p = 0.008) and down-regulation of the anti-apoptotic gene BCL2 (0.38 ± 0.19 fold, p = 0.014) in RI after cytokine exposure compared to untreated RI. In contrast, significant upregulation of BCL2 and downregulation of NF-κB1 mRNA levels were detected in RI + hAEC cocultures exposed to cytokines (Fig. 3E).

To assess islet responsiveness to glucose stimulation, static incubation assay was performed. Islets without cytokine exposure exhibited a glucose induced stimulation index of 2.1 ± 0.09 (n = 3). Exposure to cytokines significantly altered islet function with a stimulation index of 0.83 ± 0.11 (Fig. 3F). In contrast, RI co-cultured with hAECs maintained a normal insulin secretion (1.66 ± 0.02).

## Discussion

Human amniotic membrane and hAECs possess innate anti-inflammatory and immunomodulatory properties, that have been shown to be modulated by exposure to pro-inflammatory conditions [30, 34]. In this study, we confirm that exposing hAECs to inflammatory conditions increases their anti-inflammatory and immunomodulatory properties by affecting their phenotype and function. Moreover, we show that hAECs are capable to protect islets from inflammatory damage through the modulation of the inflammatory response. To the best of our knowledge, this is the first study that reports the effect of factors secreted by human hAECs on islet cell viability and function under inflammatory conditions in vitro.

**Fig. 4:**
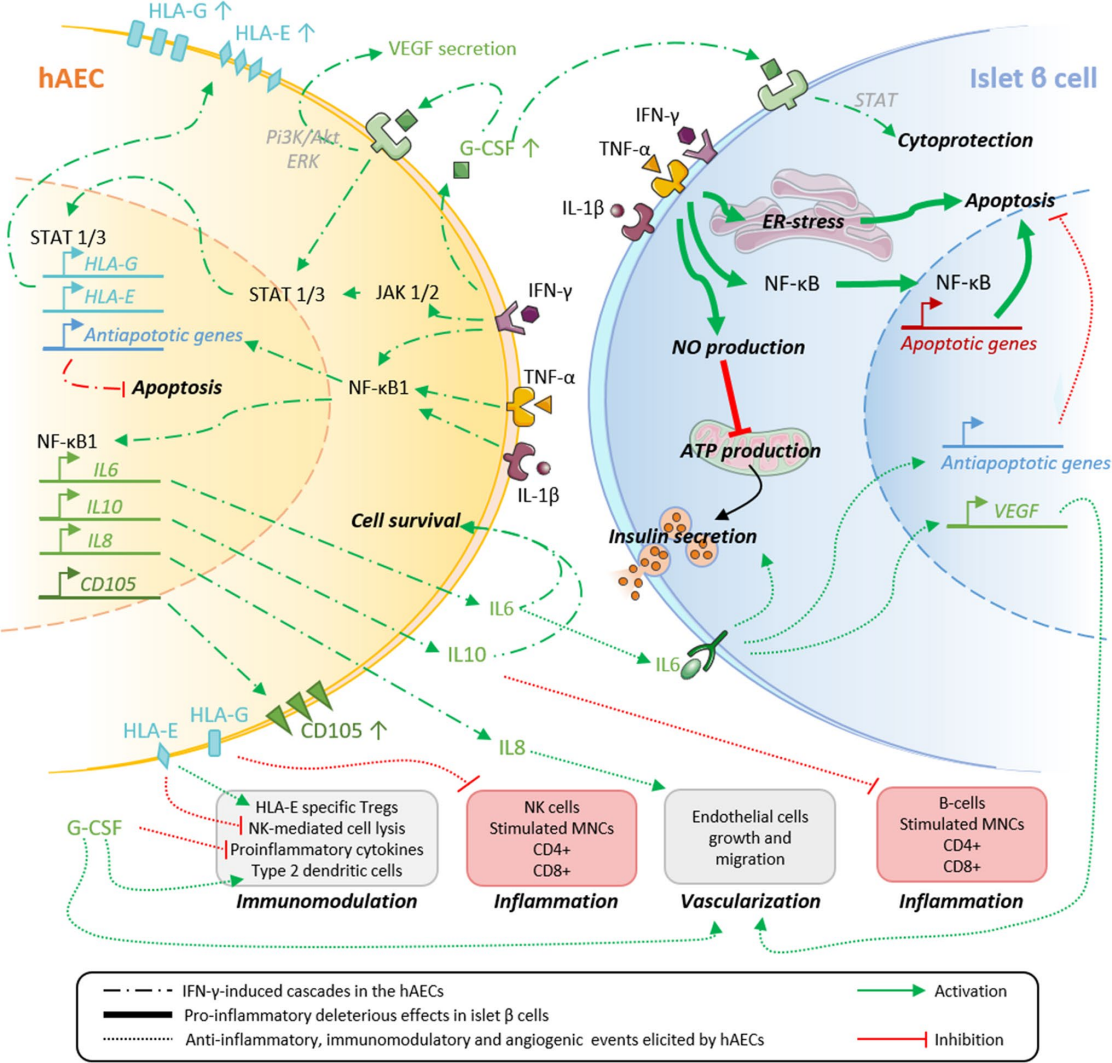
Hypothetic mechanisms behind immunomodulation and cytoprotection conferred to pancreatic islet by hAECs. Interrupted lines: Inhibitory or stimulatory cytokine-induced cascades leading to the improved immunomodulatory and anti-inflammatory properties of hAECs under pro-inflammatory conditions. Bold lines: Signaling pathways leading to proinflammatory cytokine induced islet β cell apoptosis and loss of function reported in the literature. Dotted lines: Signaling pathways triggered by the anti-inflammatory and immunomodulatory factors secreted by hAECs. Red color indicates inhibitory pathways whereas green indicates activating pathways

We have previously reported that hAECs protect islet cells from ischemic injury in vitro via HIF-1α pathway [31]. Furthermore, we showed that hAECs facilitate larger β-cell mass engraftment and improve in vivo function via acceleration of revascularization and reestablishment of cell-to-matrix contacts [31, 32]. In addition to these cytoprotective effects, hAECs have the potential to protect islets from immune destruction by inhibiting lymphocyte proliferation [49]. However, underlying mechanisms for these protective actions still need to be elucidated. Integrating the findings of this study with the relevant literature, we propose a hypothetic mechanistic model for the enhancement of anti-inflammatory and immunomodulatory properties of hAECs and the protection they confer to islets grafts (Fig. 4).

Our results showed that hAECs exposed to pro-inflammatory cytokines exhibit increased secretion of anti-inflammatory factors, in particular IL6, IL8, IL10 and G-CSF. This was associated with overexpression of the transcription factor NF-κB1, suggesting an involvement of the NF-κB1 pathway due to the activation of IFN-γ, TNF-α and IL-1β receptors, as shown in Fig. 4 (left part).

NF-κB is a multi-functional transcription factor, activated under pro-inflammatory stimuli and involved in various important biological processes including survival, inflammation, apoptosis and immune regulation [48]. At early stages of pregnancy, release of IL-1β and TNF-α from endometrial cells activates NF-κB in fetal cells, which in turn facilitates trophoblast invasion and angiogenesis. At the time of delivery, NF-κB secreted by amnion has a leading role in stimulating uterine contraction during labor by inducing in particular pro-inflammatory gene expression [37]. NF-κB is also linked to the activation of several anti-apoptotic genes, adhesion molecules and growth factors [25]. The pro-survival role of NF-κB has been attributed to the production of IL6 and IL8 [60]. Interestingly, our results demonstrating absence of apoptosis in hAECs exposed to pro-inflammatory conditions, correlated with elevation of IL6 and IL8. This can be explained by activation of NF-κB mediated anti-apoptotic signaling.

The granulocyte colony-stimulating factor (G-CSF) is a potent regulator of granulocyte production that is produced in response to the inflammatory stimuli by different hematopoietic and non-hematopoietic cells including placental tissue cells [50]. Among its many biological effects, G-CSF has a cytoprotective effect on islet cells [17]. During pregnancy, G-CSF regulates embryo implantation and development via activation of the STAT3 signaling pathway [51]. Furthermore, it was shown that G-CSF enhances MMP-2 activity and VEGF secretion in a human trophoblast cell line through activation of PI3K/Akt and Erk signaling pathways [16]. Finally, G-CSF has been shown to have modulatory effects on immune cells. In particular, it suppresses pro-inflammatory cytokines in peripheral blood mononuclear cells, induces tolerant dendritic cells (DCs), increases IL4 but reduces IFN-γ in vivo [42], and promotes tolerance to the graft in islet transplantation experiments [62].

Interestingly, we have observed increased G-CSF secretion by hAECs in response to pro-inflammatory cytokine exposure accompanied by upregulation of STAT1 and STAT3 genes, indicating involvement of G-CSF in the protective action of hAECs through the activation of STAT signaling.

Along with secretion of cytoprotective factors by primed hAECs, we observed a significant increase of immunomodulatory molecule expression. HLA-G expression increased with time, as well as in high concentrations of IFN-γ. In contrast, marked increase in HLA-E expression was detected in response to low concentrations of IFN-γ (10 U/mL). Expression of these markers is known to be regulated by pro-inflammatory conditions [18, 35]. Mechanistically, HLA-E expression is upregulated by IFN-γ, mediated by an upstream STAT1 binding site [18]. HLA-G expression is mainly regulated by IFN-γ through the JAK/STAT pathway, involving in particular STAT1 [7]. In our studies, we observed upregulation of STAT1 and STAT3, which suggests that overexpression of HLA-E and HLA-G is due to the activation of the IFNγ – JAK 1/2 – STAT1/3 pathway.

Expression of HLA-class Ib molecules, such as HLA-G and HLA-E, and anti-inflammatory molecules by hAECs exerts in turn a protective effect on islets against damages induced by inflammation. Indeed, in the immediate post-transplantation period, islets are exposed to a highly inflammatory liver microenvironment [13], where pro-inflammatory cytokines, such as IFN-γ, TNF-α and IL-1β, are largely produced in response to ischemia reperfusion injury. These factors trigger β-cell apoptosis through NF-κB activation and endoplasmic reticulum stress [9] and impair insulin secretion through an excessive nitric oxide production affecting both ATP production by the mitochondria (Fig. 4, right part, bold arrows) [11] and gap junction coupling between β-cells [14]. Infiltration of leukocytes and macrophages during the peri-transplantation period, as well as recruitment of neutrophils, macrophages, Kupffer cells and CD4 + and CD8 + lymphocytes in the later stages of engraftment also contribute to islet cell death [24].

In this context, the increased expression of HLA-G and HLA-E by hAECs under pro-inflammatory conditions appear of particular interest to protect islet grafts from inflammation-induced damage.

HLA-G and HLA-E belong to the nonclassical HLA Ib family, characterized by low polymorphism and immunomodulatory properties. HLA-G is mainly expressed by placental and embryonic tissues and participates in development of foeto-maternal tolerance [15]. In contrast, HLA-E is ubiquitously expressed and acts as an inhibitor of NK-cell driven lysis [59]. Both molecules induce immune tolerance by inhibiting DC proliferation, switching T lymphocytes to a Treg phenotype, inhibiting CD8 + and CD4 + T cells [57] and modulating the release of cytokines from mononuclear cells (MNCs) [2]. Moreover it has been shown that HLA-G and -E molecules are involved in hAEC-mediated suppression of T cell proliferation in vitro [45] (Fig. 4, bottom panels).

In addition, the immunomodulatory cytokines secreted by hAECs play a major role in suppressing inflammatory responses (Fig. 4, right panel, dashed lines). Expression of IL10 by amniotic cells has been well described, and is known to inhibit the release of pro-inflammatory mediators by monocytes and macrophages, reducing antigen presentation, and inhibiting CD4 + and CD8 + T cell differentiation and proliferation as well as B cell recruitment [57]. Increased levels of IL10 have been associated to improved islet survival and function in allotransplantation experiments while artificial upregulation of IL10 expression decreased alloreactivity to human islets and increased rat islet allograft survival [28, 55]. IL6 is known to exert anti-inflammatory actions through STAT3 activation, and has been shown to protect islets and β-cells from pro-inflammatory cytokine-induced apoptosis and loss of function in vitro*.* Moreover*,* improved survival and graft function was demonstrated after transplantation of islets pre-treated with IL6 through the overexpression of anti-apoptotic genes [8]. In addition, IL6 improves β-cell survival by stimulating autophagy and reducing cell oxidative stress [41]. Finally, IL6 exerts angiogenic effects by inducing expression of vascular endothelial growth factor (VEGF) in various cell lines [12]. Another factor overexpressed in cytokine-exposed hAECs is IL8, a neutrophil-recruiting cytokine, which is also known to promote endothelial cell proliferation [36] and angiogenesis [46] and thus may contribute to improved vascularization [31].

## Conclusion

In conclusion, our results demonstrate that anti-inflammatory and immunomodulatory potential of hAECs significantly augments when exposed to inflammatory cytokines in vitro, this in turn has a cytoprotective effect on pancreatic islets in a co-culture set-up. Taken together, this indicates that integration of hAECs in islet transplants could be a valuable strategy to: i) inhibit inflammation mediated islet damage; ii) achieve local immune protection of islets after transplantation; iii) prolong islet survival and engraftment, which currently limits the application of allogeneic islet transplantation.

## Data Availability

The data that support the findings of this study are available from the corresponding author, E.B., upon reasonable request.

## Code Availability

N/A.
